# Emergent Sand Fly–Borne Phleboviruses in the Balkan Region

**DOI:** 10.3201/eid2412.171626

**Published:** 2018-12

**Authors:** Nazli Ayhan, Remi N. Charrel

**Affiliations:** Unité des Virus Emergents, Marseille, France (N. Ayhan, R.N. Charrel); University of Florida, Gainesville, Florida, USA (R.N. Charrel)

**Keywords:** phlebovirus, sandfly fever Sicilian virus, sandfly fever Naples virus, Toscana virus, Balkan virus, sand fly, Phlebotomus, viruses, Balkan region

## Abstract

Sand fly–borne phleboviruses are associated with febrile diseases and nervous system infections in the Mediterranean basin. Sandfly fever was first reported in the Balkan Peninsula at the end of the 19th century. Since then, accumulating data show that the Balkan Peninsula, as a transboundary region between Asia and Europe, plays a major role in the emergence of vectorborne diseases in Europe. To provide an inclusive approach, we collected published data on phleboviruses in the Balkan countries and used them to evaluate the impact of these pathogens from virologic, epidemiologic, and public health perspectives. Recent findings show a high diversity of phleboviruses belonging to 3 species or serocomplexes circulating heavily in the Balkans. Focusing on undisputable human pathogens, we found direct and indirect laboratory documentation for Toscana virus, Sandfly fever Sicilian virus, and Adria virus. These data demonstrate that the Balkans are a hotspot for phleboviruses transmitted by sand flies.

Phleboviruses (genus *Phlebovirus*, family *Phenuiviridae*, order Bunyavirales) are 80–120 nm in length and display helical symmetry. Their genome consists of 3 segmented negative-sense single-stranded RNA: the large segment encodes the viral RNA polymerase (RdRp), the medium segment encodes envelope glycoproteins (Gn and Gc), and the small segment encodes nucleocapsid protein (N) and nonstructural protein (NS) (*1*,*2*). The segmented nature of the genome allows recombination and reassortment to occur with the potential to generate new viruses with distinct ancestors (*3*,*4*). Segment reassortment in Bunyavirales has been reported with increasing frequency, especially in the genus *Orthobunyavirus* (*5*). Specifically, reassortant viruses have been described in both Candiru and Rift Valley fever species (*5*,*6*).

Two sand fly–borne phleboviruses in the Old World were historically associated with cases of sandfly fever: Sicilian virus and Naples virus (*7*). Later, Naples virus was renamed sandfly fever Naples virus (SFNV), which is included in the *Sandfly fever Naples virus* species*.* Likewise, Sicilian virus was renamed sandfly fever Sicilian virus (SFSV), which is still a tentative species. SFSV and SFNV are both responsible for sandfly fever, a self-limiting but incapacitating febrile illness. Toscana virus (TOSV), discovered in 1971, was incriminated as causing central and peripheral nervous system infections in 1983 (*8*,*9*). TOSV can cause aseptic meningitis and meningoencephalitis (*9*–*12*), as well as a number of other manifestations affecting the central and peripheral nervous system. These viruses are transmitted via bites of *Phlebotomus* spp. sand flies.

Data concerning the geographic distribution of SFSV, SFNV, and TOSV have drastically increased during the past 2 decades, resulting in a more accurate cartography of their presence in the Mediterranean basin, the Middle East, and central Asia (*12*–*16*).

The Balkan Peninsula is a principal region for sandfly fever. It is located in southeastern Europe, and consists of Slovenia, Croatia, Bosnia-Herzegovina, the Republic of Macedonia, Albania, Bulgaria, Greece, Montenegro, Romania, Serbia, and Kosovo. The Balkan region is composed of 3 very different natural entities: the Adriatic littoral in the southwest, the Pannonian plain in the northeast, and a broad expanse of mountainous regions in between. The first record of sandfly fever originated in Bosnia-Herzegovina at the end of the 19th century (Technical Appendix Table). During World War I and World War II, sandfly fever affected great numbers of soldiers in the region (*17*,*18*) (Technical Appendix Table). In addition to historical data, recent reports show the activity of several novel viruses with severe human infections. We reviewed all the published data for sand fly–borne phleboviruses in the Balkan Peninsula to provide a comprehensive view of the current situation and of the public health effect on humans and vertebrate animals in the region.

## Methods

We searched global web-based resources (PubMed [www.ncbi.nlm.nih.gov/pubmed], Google Scholar [https://scholar.google.com/], and Web of Science [https://isiknowledge.com]) to collect all the sand fly–borne phlebovirus data from the Balkan region. In addition, we investigated libraries and other national resources to identify books and conference reports that are not accessible on web-based resources. We used the keywords “sand fly,” “Phlebovirus,” “Bunyaviridae,” “Phenuiviridae,” “sand fly fever,” “pappataci fever,” “three-day fever,” “sandfly fever,” “Toscana virus,” “Sicilian virus,” “Naples virus,” “SFSV,” and “SFNV” matched with “Balkan,” “Balkan Peninsula,” “Yugoslavia,” “Slovenia,” “Croatia,” “Bosnia-Herzegovina,” “Macedonia,” “Republic of Macedonia,” “Former Yugoslav Republic of Macedonia,” “FYROM,” “RoM,” “Albania,” “Bulgaria,” “Greece,” “Montenegro,” “Romania,” “Moldova,” “Serbia,” and “Kosovo” for the research. After gathering all the data, we discarded the irrelevant publications. We put the collected data in order based on country, year, and the phlebovirus species complex.

**Figure 1:**
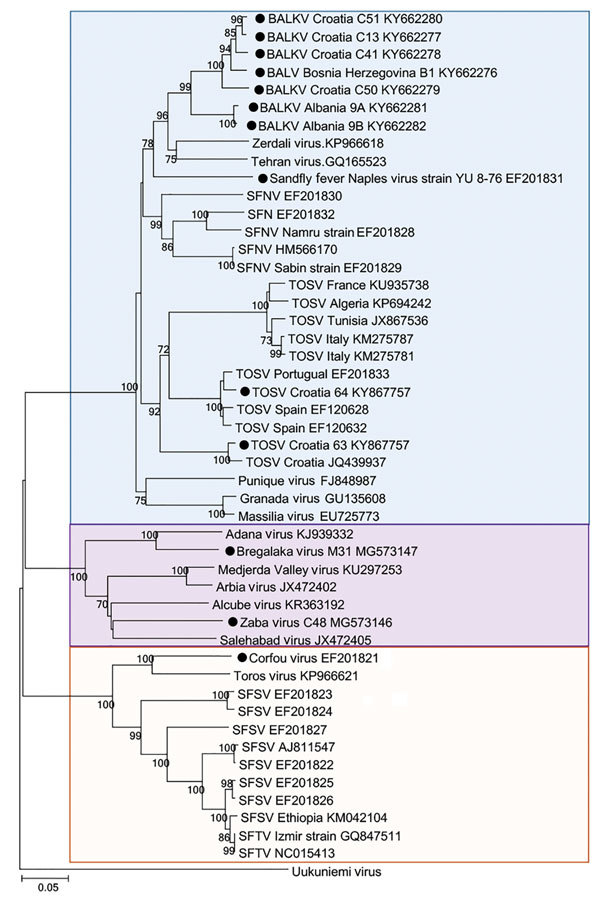
Phylogenetic relationships between sand fly–borne phleboviruses in the Old World based on 589 nt partial nucleoprotein sequence. Phylogenetic tree was constructed using the neighbor-joining method with MEGA software version 6 (http://www.megasoftware.net). Black circles indicate viruses identified in the Balkan region. Boxes (top to bottom) include all viruses belonging to the *Sandfly fever Naples virus* species, viruses belonging to the *Salehabad phlebovirus* species, viruses belonging to the *Sandfly fever Sicilian* and *Corfou* tentative virus species. The robustness of each node was tested by 1,000 bootstrap replicates. GenBank accession numbers are provided. Scale bar indicates nucleotide substitutions per site.

We obtained all the accessible virus sequences from Balkan countries from GenBank. We aligned 589 nt partial nucleoprotein sequences and analyzed them with MEGA software version 6 (https://www.megasoftware.net/*)*. We constructed a phylogenetic tree using the neighbor-joining method (Figure 1) and tested the robustness of each node by 1,000 bootstrap replicates.

## Results

We collected 51 published articles: 2 articles from Albania, 7 from Bosnia-Herzegovina, 11 from Croatia, 17 from Greece, 5 from Kosovo, 1 from Republic of Macedonia, and 7 from Serbia (Technical Appendix Table). One reference from Bulgaria was not available (Technical Appendix Table). We found no published data from Montenegro and Romania. Most of the references included data concerning seroprevalence studies conducted in humans or animals (Technical Appendix Table). Several articles reported results about either virus characterization or case reports/outbreak investigations (*19*–*25*) (Technical Appendix Table).

### Historical Data on Phleboviruses in the Balkans

Alois Pick made a clinical description of sandfly fever in Bosnia-Herzegovina military barracks from foreign soldiers at the end of the 19th century (Technical Appendix Table). Pick, an Austro-Hungarian military doctor working in Trebinje (Herzegovina), characterized the syndrome observed in cases of sandfly fever (*26*). Sandfly fever was observed both in local populations and in visitors, specifically foreign soldiers. In 1904, Taussig noticed the presence of “pappataci” sand flies in army barracks in Herzegovina and conducted a large clinical and epidemiologic study in the region (*27*). The “endemic disease” emerged only in places where sand flies were present. Subsequently, the causative agent was discovered as a filterable agent (virus) that used pappataci sand flies as a vector (*18*). 

During World War II, sandfly fever affected great numbers of foreign soldiers in all Mediterranean region and Balkan countries during the summer seasons, when sand fly activity peaks (*17*). The disease was called phlebotomus fever, pappataci fever, or three-day fever. In 1937, a massive outbreak occurred in Athens, Greece (Technical Appendix Table). After World War II, sandfly fever epidemics were recorded in Belgrade, Serbia, affecting thousands of persons and then expanding into other regions of the Balkans (*27*) (Technical Appendix Table). Although these articles could be the first record of sandfly fever based on clinical and epidemiologic grounds, there is no scientific evidence to demonstrate whether the disease described in the articles was sandfly fever caused by phlebovirus.

The seminal seroprevalence study using a neutralization assay, by Tesh et al. in 1976, showed that SFNV and SFSV had circulated and were likely to continue to infect human populations in the tested regions (Technical Appendix Table). Also in 1976, Gligić et al. isolated a strain of SFNV (Yug Bogdanovac virus strain Yu 8/76) from *P. perfiliewi* sand flies in the Dobrič region of Serbia. Other strains of SFNV and SFSV were also isolated in Serbia from *P. pappatasi* sand flies, but no accessible sequence data are available (*24*). At the time of isolation, SFNV and SFSV identification was done using mouse hyperimmune ascitis fluid for neutralization assays and acetone sucrose antigens for complement fixation tests. SFNV strain YU-8-76 is available in the Yale University catalog, now stored at the University of Texas Medical Branch at Galveston. Partial sequence of this strain has been determined and confirmed the strain as belonging to the *Sandfly fever Naples virus* species (*28**,**29*).

In 1985, Corfou virus, closely related to but distinct from SFSV, was isolated from *P. neglectus* sand flies collected in the island of Corfou, Greece (Technical Appendix Table). Corfou and SFSV can be distinguished only by neutralization assays, unlike other serologic assays (ELISA, hemagglutination inhibition [HI], indirect immunofluorescence [IIF], complement fixation [CF]). Several studies have confirmed the presence of antibodies against both SFNV and SFSV in several areas of the Balkans (*20*) (Technical Appendix Table).

### Toscana Virus in the Balkan Region

In 1993, a German traveler was infected with TOSV after visiting Athens; diagnosis was established from immunofluorescence serology results, and it is therefore classified as a probable case rather than a laboratory-confirmed case (Technical Appendix Table). Recent serologic studies have provided evidence of TOSV presence in Bosnia-Herzegovina, Kosovo, Croatia, and Greece (Technical Appendix Table). Several human cases documented serologically as TOSV infections have been reported in Greece (*23*) (Technical Appendix Table).

**Figure 2:**
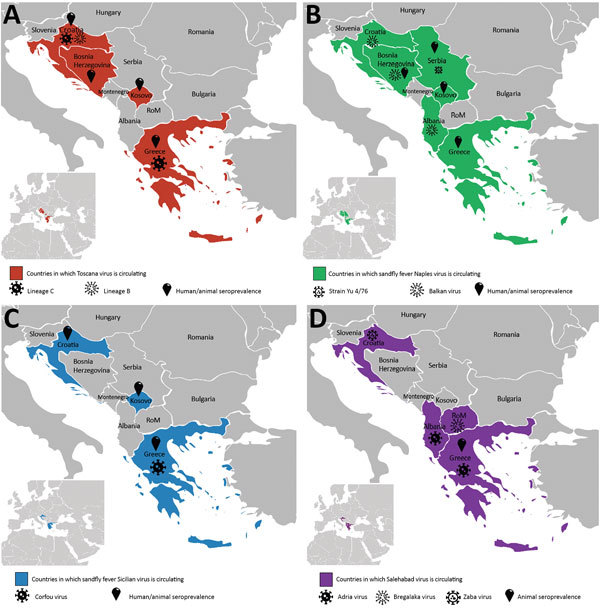
Current distribution of sand fly–borne phleboviruses in the Balkan region. A) Toscana virus, B) sandfly fever Naples virus, C) sandfly fever Sicilian virus, and D) Salehabad virus. Pictograms refer to virus isolation or sequence data demonstrating the presence of the virus in that area. Human/animal seroprevalence refers to studies reporting the presence of specific antibodies against the virus mentioned in the panel.

In Croatia, TOSV RNA was detected in the cerebrospinal fluid of a patient infected with meningitis; sequence analysis showed that he was infected with a strain belonging to a genetic lineage that had not been previously recognized (subsequently named lineage C), which was clearly distinct from lineages A and B (Technical Appendix Table). Subsequently, TOSV lineage C was detected from a patient in Greece (Technical Appendix Table); unfortunately, the virus was not isolated in both cases and therefore only partial sequence data are available. Later, sequences obtained from *P. neglectus* sand flies confirmed the presence of lineage C TOSV but also showed that lineage B TOSV was present and that both genetic types were sympatric in Croatia (Figure 2; Technical Appendix Table). 

### New Phleboviruses Identified from Partial Genomic Sequences

A novel phlebovirus, Adria virus, was detected in 2 pools of sand flies collected in Albania in 2005 (Technical Appendix Table). Adria virus is most closely related to Arbia virus, which was isolated in Italy (*8*); both belong to the *Salehabad phlebovirus* species. Adria virus RNA was detected in the blood of a 2.5-year-old patient with febrile seizures in Greece (Technical Appendix Table). This evidence showed that a virus within the *Salehabad phlebovirus* species could be associated with human disease (Figure 2). 

Balkan virus (BALKV) was detected from 2 pools of *P. neglectus* sand flies in Albania in 2014, 1 pool from Bosnia-Herzegovina in 2014–15, and 4 pools from Croatia in 2015 (Technical Appendix Table). Sequence data analysis showed that BALKV belongs to the *Sandfly fever Naples virus* species, where it clusters with subgroup I together with Tehran, Zerdali, Fermo, and SFNV YU 8–76 viruses respectively discovered in Iran, Turkey, Italy, and Serbia (*30*–*32*) (Figure 2; Technical Appendix Table). 

### New Phleboviruses Identified from Complete Genomic Sequences

Bregalaka virus (BREV) was isolated in *P. perfiliewi* sand flies from the Republic of Macedonia in 2015. Sequence analysis demonstrated that BREV is most closely related to Adana virus, which was isolated in Turkey from field-collected sand flies in 2012. In Croatia, Zaba virus (ZABAV) was isolated from *P. neglectus* sand flies. ZABAV is most closely related to Adria virus and Salehabad virus. Both BREV and ZABAV belong to the *Salehabad phlebovirus* species (Technical Appendix Table).

### Human and Animal Exposure to Phleboviruses

SFSV and SFNV are both responsible for a febrile illness that is self-limited but incapacitating, with signs that are commonly observed in arboviral diseases, such as fever, headache, malaise, photophobia, myalgia, and retroorbital pain. From a clinical perspective, it is impossible to distinguish SFNV from SFSV infections, and also to discriminate between SFNV/SFSV and other arboviral infections. As mentioned previously, historic records were based on clinical and epidemiologic evidence, but virological documentation was lacking for studies before the 1950s (Figure 2; Technical Appendix Table). 

Although SFSV and SFNV infections are clinically indistinguishable from each other, they are caused by genetically and antigenically different viruses. Infection with SFNV does not induce cross-protection against SFSV and vice versa (*33*). As mentioned previously, neutralization test is the only technique that permits undisputable identification at the specific and intraspecific levels. Other techniques, such as ELISA, CF, HI, and IFA, which are prone to cross reactions, cannot achieve unambiguous identification at the intraspecific or at the interspecific level.

Seroprevalence studies conducted in the Balkans from 1976 onward have described antibodies in human populations confirming exposure to several phleboviruses transmitted by sand flies. Complement-fixation tests showed antibodies against SFNV in Bosnia-Herzegovina (Technical Appendix Table), and HI tests showed antibodies against SFNV and SFSV in the islands of Croatia (Table; Technical Appendix Table). In Greece, neutralizing antibodies against SFNV and SFSV were described; 36% of persons >30 of age showed positive results for SFSV, and 13% showed positive results for SFNV. Persons <30 years of age had much lower rates, suggesting that the antimalarial campaign had drastically reduced the sand fly population and therefore the exposure to viruses transmitted by sand flies (Technical Appendix Table). Presence of neutralizing antibodies against SFSV showed wide circulation (71.9%) in mainland and island regions of Greece in dogs used as sentinel animals; in the same study, TOSV and Arbia virus neutralizing antibodies were also found at lower rates: 4.4% for TOSV and 2.6% for Arbia virus (Technical Appendix Table). In Kosovo, 9.6% of the 104 human serum samples tested were positive for neutralizing antibodies against SFSV and 27.9% of the serum samples were positive for neutralizing antibodies against SFNV (Technical Appendix Table). With the same technique, 58.5% of cattle and 22.2% of sheep were positive (Technical Appendix Table). CF antibodies were found for SFNV in 19.4% of human serum samples in Serbia (Technical Appendix Table). TOSV was discovered in 1971, but it was identified as a human pathogen 12 years later, which prevented early inclusion in the seroprevalence studies; thus, almost no data exist for TOSV before the 1990s.

**Table:** Characteristics of sand fly–borne phleboviruses in the Balkan region

Virus	Taxonomy	Source	Virus isolation	Distribution	Probable vector species	Human/animal infections
Sand fly fever Naples virus*	*Sandfly fever Naples virus* species	Field-collected sand flies	Yes, sand fly pools	Serbia	*P. perfiliewi*	Yes
Corfu virus	*Sandfly fever Sicilian virus* species†	Field-collected sand flies	Yes, sand fly pools	Greece	*P. neglectus*	Probable
Adria virus	*Salahabad virus* species	Field-collected sand flies, patient blood	No, partial L segment sequence available	Albania, Greece	*Phlebotomus* spp.	Yes
Balkan virus	*Sandfly fever Naples virus* species	Field-collected sand flies	No, partial L and S segment sequences available	Albania, Bosnia-Herzegovina, Croatia	*P. neglectus*	Unknown
Bregalaka virus	*Salahabad virus* species	Field-collected sand flies	Yes, sandfly pools	Republic of Macedonia	*P. perfiliewi*	Unknown
Zaba virus	Salahabad virus species	Field-collected sand flies	Yes, sandfly pools	Croatia	*P. neglectus*	Unknown
Toscana virus	Sandfly fever Naples virus species	Field collected sand-flies, CSF	No, partial L and S segment sequences available	Croatia, Greece	*P. neglectus*	Yes

*Yug Bogdanovac virus strain Yu 4/76. †Tentative species.

Recent data have confirmed the circulation of TOSV and associated human cases in Kosovo, Greece, and Croatia. In Croatia, 2 risk factors were associated with TOSV positive serology: living on an island and age (Technical Appendix Table). Possible presence of TOSV was assessed in Bosnia-Herzegovina through immune-line assays (Technical Appendix Table). TOSV neutralizing antibodies were detected in cats and dogs in Greece and in cattle and sheep in Kosovo (Figure 2; Technical Appendix Table). 

## Discussion

Sand fly–borne diseases are widespread in the Balkan region because of the favorable climate and socioeconomic conditions in that area. After the first record of sandfly fever in Bosnia-Herzegovina at the end of the 19th century (Technical Appendix Table), several outbreaks occurred in the whole Balkan region. Epidemics of sandfly fever and leishmaniasis prompted faunistic and ecologic investigations of sand flies from 1947 through the 1970s (*33*). The number of studies on sand fly fauna has decreased since that time because of the decline in recorded sandfly fever cases. For some Balkan countries, almost nothing is known about sand fly distribution; when data are available, they are too old to reflect the current situation accurately. The collapse of the former Yugoslavia and subsequent armed conflicts have also contributed to the lack of sustained studies on sand fly–borne pathogens in this region. However, recent data show that the Balkan region is still a major hotspot for arboviral diseases. 

Most virus studies are based on serosurveillance. The seminal neutralization-based seroprevalence study, performed by Tesh et al. in the 1970s, identified antibodies against SFNV and SFSV in human populations from Croatia, Greece, and Kosovo (Technical Appendix Table). Successive studies confirmed the presence of antibodies against phleboviruses in most parts of the Balkans, and recent serologic studies show the circulation of TOSV in Bosnia-Herzegovina, Kosovo, Croatia, and Greece (Technical Appendix Table).

SFNV Yu 8/76 was the first phlebovirus isolated in the Balkans (Technical Appendix Table). It was isolated from *P. perfiliewi* sand flies; before this finding, other viruses had been isolated from *P. papatasi* sand flies, which were believed to be the unique vector competent for SFNV and SFSV (*17*). The recent discovery of BALKV should stimulate studies to address possible human pathogenicity (Technical Appendix Table). Recent evidence for the presence of at least 2 different lineages of TOSV calls for studies to measure its involvement in summer meningitis and other neurologic infections, as were performed in Italy after the discovery of TOSV neurotropism.

Corfou virus was isolated from *P. major* sand flies in the eponymous Greek island (Technical Appendix Table). Although Corfou/SFSV circulation was assessed by seroprevalence studies, Corfou virus remains the only SFSV-like virus isolated in the Balkans.

Adria virus was the first member of the *Salehabad* virus complex to be associated with human disease. Because of this finding, and in light of the newly discovered viruses within this species (BREV and ZABAV), future actions should be directed at implementing direct and indirect diagnosis of *Salehabad phlebovirus* species in clinical microbiology laboratories to better understand their potential public health impact.

The fall of communism, the breakup of the former Yugoslavia, and the following civil war and other climatic and environmental changes resulted in an increase of zoonotic infections that emerged or reemerged in the Balkans (*28*). As previously suggested for North Africa and in Turkey, it is time to organize systematic testing of patients with CNS infections or unexplained febrile illness for such viruses in clinical microbiology laboratories in hospitals (*35*–*37*). 

In summary, when historical and recent data are compiled, it appears that the Balkan region is a hotspot for viruses transmitted by sand flies, including those that cause diseases in humans. The variety of different viruses is higher than in other regions that were investigated, and certain areas display sympatric circulation of several viruses. Circulation of these viruses must be assessed by studies conducted in human populations and vertebrates, and diagnosis of human infections caused by sand fly–borne viruses must now be implemented using molecular and serologic tools in clinical microbiology laboratories.

Technical Appendix Results of literature search for study of sand fly–borne phleboviruses in the Balkan region.
