# Supplementary material for: Emergent Sand Fly–Borne Phleboviruses in the Balkan Region
Source: Emerg Infect Dis. 2018 Dec;24(12):2324–30. doi: 10.3201/eid2412.171626 (PMC6256412; doi:10.3201/eid2412.171626)
Supplement: Technical Appendix — Results of literature search for study of sand fly–borne phleboviruses in the Balkan region. [file 17-1626-Techapp-s1.pdf]

Article DOI: https://doi.org/10.3201/eid2412.171626

Emergent Sand Fly–Borne Phleboviruses in the Balkan Region

Technical Appendix

Technical Appendix Table. Scientific sources of the data used in the study of sand fly–borne viruses in the Balkan region*

Country	Study design	Virus species	Region	Information	Seroprevalence	Assays	Reference
Albania	Vector surveillance	Adria virus	Kruje and Lezhe	Viral detection from *Phlebotom* spp.		PCR	(*5*)
	Vector surveillance	Balkan virus	Kruje	Virus detection from *P. neglectus*		PCR	(*6*)
Bosnia-Herzegovina	Case reports	Unidentified phlebovirus	Herzegovina	Case reports from military barracks		Clinical diagnosis	(*2*,*3*)
	Serosurveillance	SFNV		Human seroprevalence	62.1%	CF	(*33*)
	Serosurveillance	SFNV		Human seroprevalence	?	CF	(*7*)
	Serosurveillance	SFNV		Human seroprevalence	15.7 %	HI	(*11*,*12*)
	Serosurveillance	SFSV		Animal seroprevalence (sheep, cattle)	13.5%–31.2%	HI	(*11*,*12*)
	Serosurveillance	TOSV	Sarajevo	Human seroprevalence, 2006	12.5%	IgG+IgM IT	(*4*)
				2007	9.38%		
				2008	10.71%		
	Vector surveillance	Balkan virus	Sovici	Virus detection from *P. neglectus*		PCR	(*8*)
Croatia	Serosurveillance	Unidentified phlebovirus	Croatian littoral	Human seroprevalence	22.6%	HI	(*11*,*12*)
			Mljet Island		51.4%		
	Serosurveillance	Unidentified phlebovirus	Brac	Human seroprevalence	46.4%	HI	(*1*)
			Hvar		33.9%		
			Korcula		26.0%		
	Serosurveillance	SFNV	Brac, Dalmatia Province	Human seroprevalence	57.6%	PRNT	(*9*)
	Serosurveillance	SFNV		Human seroprevalence	23.6%	HI	(*13*)
	Serosurveillance	SFNV	Mljet Island	Human seroprevalence	51.4%	VNT	(*10*)
	Serosurveillance	SFSV	Brac, Dalmatia Province	Human seroprevalence	15.6%	PRNT	(*9*)
	Serosurveillance	TOSV	Adriatic coast	Human seroprevalence	33.6%	IT	(*14*)
			Adriatic islands		53.9%		
			Croatian mainland		6.1%		
	Case report	TOSV	Split	Case report and viral detection from CSF		PCR	(*13*)
	Vector surveillance	TOSV	Vidonje	TOSV Lin B and Lin C detection from *P. neglectus*		PCR	(*15*)

Country	Study design	Virus species	Region	Information	Seroprevalence	Assays	Reference
	Vector surveillance	Balkan virus	Duba, Vidonje	Virus detection from *P. neglectus*		PCR	(*8*)
	Vector surveillance	Zaba virus	Vidonje	Virus isolation from *P. neglectus*		PCR, cell culture, sequencing	Unpub. data
Greece	Case reports	Unidentified phlebovirus	Athens	Sandfly fever outbreak in 1937		Clinical diagnosis	(*16*)
	Serosurveillance	SFNV		Human seroprevalence >30 years	36%	PRNT	(*17*)
				Human seroprevalence <30 years	4%		
	Serosurveillance	SFNV	Athens	Human seroprevalence	24.7%	PRNT	(*9*)
			Malta (Crete)		13.1%		
	Serosurveillance	SFNV	25 different locations	Human seroprevalence	16.7%	PRNT	(*18*)
	Serosurveillance	SFSV		Human seroprevalence <30 years	13%	PRNT	(*17*)
	Serosurveillance	SFSV	Athens	Human seroprevalence	8.5%	PRNT	(*9*)
	Serosurveillance	SFSV	25 different locations	Human seroprevalence	2.0%	PRNT	(*18*)
	Serosurveillance	SFSV	12 different locations	Animal seroprevalence (dog)	50.7–84.9%	VNT	(*27*)
	Case-based surveillance	TOSV		Case report (German traveler)		IFA	(*20*)
	Case-based surveillance	TOSV	Thessaloniki	Case report (8-year-old boy)		IgG	(*21*)
	Serosurveillance	TOSV	Corfu	Human seroprevalence	51.7%	ELISA, IFA	(*24*)
			Cephalonia		39%		
	Serosurveillance	TOSV	North Greece	Human seroprevalence	11.26%	VNT	(*22*)
	Serosurveillance	TOSV	Islands	Human seroprevalence	21%	VNT	(*23*)
	Case report	TOSV	Trikala	Case report and Virus detection from CSF		PCR	(*25*)
	Case report	TOSV	Serres	A probable case with symptoms		IgG + IgM IFA	(*26*)
	Serosurveillance	TOSV		Animal seroprevalence (dog)	0–15.4%	VNT	(*27*)
	Vector surveillance	Corfu virus	Corfu Island	Virus isolation from *P. neglectus*		PCR, cell culture	(*19*)
	Case report	Adria virus	Thessaloniki	Case report and Virus detection from blood		PCR	(*21*)
	Serosurveillance	Arbia virus	12 different locations	Animal seraprovalence (dog)	2.6%	VNT	(*27*)
Kosovo	Serosurveillance	Unidentified Phlebovirus	Kamenica	Human seroprevalence	27.4%	PRNT	(*28*)
	Serosurveillance	SFNV		Human seroprevalence	27.9%	PRNT	(*9*)
	Serosurveillance	SFNV	Kamenica	Human seroprevalence	22.10%	PRNT	(*28*)
	Serosurveillance	SFNV	Peja	Human seroprevalence	1.00%	PRNT	(*29*)
	Serosurveillance	SFSV		Human seroprevalence	9.6%	PRNT	(*9*)
	Serosurveillance	SFSV	12 different locations	Animal seroprevalence (sheep, cattle)	22.2%–58.52%	VNT	(*30*)

Country	Study design	Virus species	Region	Information	Seroprevalence	Assays	Reference
	Serosurveillance	TOSV	Peja	Human seroprevalence	5.5%	ELISA	(*29*)
	Serosurveillance	TOSV	Peja	Human seroprevalence	0.5%	PRNT	(*29*)
	Serosurveillance	TOSV	12 different locations	Animal seroprevalence (sheep, cattle)	1.96%–5.14%	VNT	(*30*)
Former Yugoslav Republic of Macedonia	Vector Surveillance	Bregalaka Virus	Kezovica, Suvo Grlo	Virus isolation from *P. perfiliewi*		PCR, cell culture, sequencing	Unpub. data
Serbia	Case reports	Unidentified phlebovirus	East Serbia, Belgrade	Sandfly fever outbreak in 1946		Clinical diagnosis	(*31*)
	Case reports	Unidentified phlebovirus	Banat	Sandfly fever outbreak in 1946		Clinical diagnosis	(*32*)
	Case reports	Unidentified phlebovirus	South Serbia	Sandfly fever outbreak in 1950		Clinical diagnosis	(*32*)
	Vector surveillance and serosurveillance	SFNV (Yug Bogdanovac virus strain Yu 8/76)	Dobrič	Virus isolation from *P. perfiliewi*		PCR, animal experiment	(*33*)
				Human seroprevalence	4.16%	CF	
				Animal seroprevalence	12.96%	CF	
	Serosurveillance	SFNV		Human seroprevalence	19.4%	CF	(*34*)

*CF, complement fixation; CSF, cerebrospinal fluid; HI, hemagglutination inhibition test; IFA, indirect immunofluorescence assay; IT, immunoassay test; PRNT, plaque reduction neutralization test; SFNV, sandfly virus Naples virus; TOSV, Toscana virus; VNT; virus neutralization test

References

1. Vesenjak-Hirjan J, Punda-Polić V, Dobe M. Geographical distribution of arboviruses in Yugoslavia. J Hyg Epidemiol Microbiol Immunol. 1991;35:129–40.

2. Pick A. On the pathology and therapy of a peculiar endemic 1860 disease form [in German]. Wien Med Wochenschr. 1886;33:1141–5.

3. Pick A. Contributions to the pathology and therapy of a peculiar 1862 disease form (gastro-enteritis climatica) [German]. Prager Med Wschr. 1887;12:364.

4. Hukić M, Salimović-Besić I. Sandfly-pappataci fever in Bosnia and Herzegovina: the new-old disease. Bosn J Basic Med Sci. 2009;9:39–43. http://dx.doi.org/10.17305/bjbms.2009.2854

5. Papa A, Velo E, Bino S. A novel phlebovirus in Albanian sandflies. Clin Microbiol Infect. 2011;17:585–7. http://dx.doi.org/10.1111/j.1469-0691.2010.03371.x

6. Ayhan N, Velo E, de Lamballerie X, Kota M, Kadriaj P, Ozbel Y, et al. Detection of *Leishmania infantum* and a Novel Phlebovirus (Balkan Virus) from Sand Flies in Albania. Vector Borne Zoonotic Dis. 2016;16:802–6. http://dx.doi.org/10.1089/vbz.2016.2002

7. Terzin AL, Matuka S, Fornazarić MR, et al. Antibodies against some arboviruses and against the Bedsonia antigen in sera of men, sheep and cattle in Bosnia and Herzegovina. Acta Medica Yugoslavica XVI: Fasc. 1962;3–4:301–17.

8. Ayhan N, Alten B, Ivovic V, Dvořák V, Martinkovic F, Omeragic J, et al. Direct evidence for an expanded circulation area of the recently identified Balkan virus (Sandfly fever Naples virus species) in several countries of the Balkan archipelago. Parasit Vectors. 2017;10:402. http://dx.doi.org/10.1186/s13071-017-2334-y

9. Tesh RB, Saidi S, Gajdamovič SJA, Rodhain F, Vesenjak-Hirjan J. Serological studies on the epidemiology of sandfly fever in the Old World. Bull World Health Organ. 1976;54:663–74.

10. Punda-Polić V, Calisher CH, Vesenjak-Hirjan J. Neutralizing antibodies for sandfly fever Naples virus in human sera on the island of Mljet. Acta Med Iugosl. 1990;44:15–20.

11. Vesenjak-Hirjan J. Galinović -Weisglass M, Urlić V. Occurrence of arboviruses in the middle and the south Adriatic (Yugoslavia). Arboviruses in the Mediterranean countries. Zbl Bakt. 1980;2:303–10.

12. Vesenjak-Hirjan J. Arboviruses in Yugoslavia. Arboviruses in the Mediterranean countries. In Vesenjak-Hirjan, J. (Ed.). Stuttgart-New York, Gustav Fischer Verlag. 1980;165–177.

13. Punda-Polić V, Mohar B, Duh D, Bradarić N, Korva M, Fajs L, et al. Evidence of an autochthonous Toscana virus strain in Croatia. J Clin Virol. 2012;55:4–7. http://dx.doi.org/10.1016/j.jcv.2012.06.006

14. Punda-Polić V, Jerončić A, Mohar B, Šiško Kraljević K. Prevalence of Toscana virus antibodies in residents of Croatia. Clin Microbiol Infect. 2012;18:E200–3. http://dx.doi.org/10.1111/j.1469-0691.2012.03840.x

15. Ayhan N, Alten B, Ivovic V, Martinkovic F, Kasap OE, Ozbel Y, et al. Co-circulation of two lineages of Toscana virus in Croatia. Front Public Health. 2017;5:336. http://dx.doi.org/10.3389/fpubh.2017.00336

16. Alivisatos GP, Pagonis A, Triantaphyllou T. On the three-day fever epidemic of 1935 in Athens and its environs [French]. Bull Off Int Hyg Publ. 1936;28:2146.

17. Tesh RB, Papaevangelou G. Effect of insecticide spraying for malaria control on the incidence of sandfly fever in Athens, Greece. Am J Trop Med Hyg. 1977;26:163–6. http://dx.doi.org/10.4269/ajtmh.1977.26.163

18. Antoniadis A, Alexiou-Daniel S, Malisiovas N, et al. Seroepidemiological survey for antibodies to arboviruses in Greece. Arch Virol. 1990;Suppl 1:277–85.

19. Rodhain F, Madulo-Leblond G, Hannoun C, Tesh RB. Le virus Corfou: Un nouveau *Phlebovirus* isolé de phlébotomes en Grèce. Ann Inst Pasteur Virol. 1985;136:161–6. http://dx.doi.org/10.1016/S0769-2617(85)80042-3

20. Dobler G, Treib J, Haass A, Frösner G, Woesner R, Schimrigk K. Toscana virus infection in German travellers returning from the Mediterranean. Infection. 1997;25:325. http://dx.doi.org/10.1007/BF01720413

21. Anagnostou V, Pardalos G, Athanasiou-Metaxa M, Papa A. Novel phlebovirus in febrile child, Greece. Emerg Infect Dis. 2011;17:940–1. http://dx.doi.org/10.3201/eid1705.101958

22. Anagnostou V, Papa A. Prevalence of antibodies to phleboviruses within the sand fly fever Naples virus species in humans, northern Greece. Clin Microbiol Infect. 2013;19:566–70. http://dx.doi.org/10.1111/j.1469-0691.2012.03957.x

23. Anagnostou V, Papa A. Seroprevalence of Toscana virus among residents of Aegean Sea islands, Greece. Travel Med Infect Dis. 2013;11:98–102. http://dx.doi.org/10.1016/j.tmaid.2012.11.006

24. Papa A, Andriotis V, Tzilianos M. Prevalence of Toscana virus antibodies in residents of two Ionian islands, Greece. Travel Med Infect Dis. 2010;8:302–4. http://dx.doi.org/10.1016/j.tmaid.2010.09.002

25. Papa A, Paraforou T, Papakonstantinou I, Pagdatoglou K, Kontana A, Koukoubani T. Severe encephalitis caused by Toscana virus, Greece. Emerg Infect Dis. 2014;20:1417–9. http://dx.doi.org/10.3201/eid2008.140248

26. Papa A, Mallias J, Tsergouli K, Markou F, Poulou A, Milidis T. Neuroinvasive phlebovirus infection in Greece: a case report. Intervirology. 2014;57:393–5. http://dx.doi.org/10.1159/000363505

27. Alwassouf S, Christodoulou V, Bichaud L, Ntais P, Mazeris A, Antoniou M, et al. Seroprevalence of sandfly-borne phleboviruses belonging to three serocomplexes (sandfly fever Naples, sandfly fever Sicilian and Salehabad) in dogs from Greece and Cyprus using neutralization test. PLoS Negl Trop Dis. 2016;10:e0005063. http://dx.doi.org/10.1371/journal.pntd.0005063

28. Salja S, Imami O, Galinovic-Weisglass M, et al. Arbovirus infections in the region of Kosovska Kamenica (Yugoslavia). Stuttgart, New York: Gustav Fischer Verlag; 1980. p. 285–9.

29. Venturi G, Marchi A, Fiorentini C, Ramadani N, Quaglio G, Kalaveshi A, et al. Prevalence of antibodies to phleboviruses and flaviviruses in Peja, Kosovo. Clin Microbiol Infect. 2011;17:1180–2. http://dx.doi.org/10.1111/j.1469-0691.2010.03445.x

30. Ayhan N, Sherifi K, Taraku A, Bërxholi K, Charrel RN. High rates of neutralizing antibodies to Toscana and sandfly fever Sicilian viruses in livestock, Kosovo. Emerg Infect Dis. 2017;23:989–92. http://dx.doi.org/10.3201/eid2306.161929

31. Karakešević B. The first epidemic of "pappataci fever" in the territory of Serbia [in Serbian]. Vojno sanitetski pregled. 1947;9;224–8.

32. Simić Ć. The apparent appearance of "pappataci fever" in Serbia and Banat [in Serbian]. Glas Srpske akademije nauka CCIV. Odjeljenje medicinskih nauka. 1951;4:143–152.

33. Gligić A, Miščević Z, Živković V, et al. Characteristic morphological and immunological features of viruses newly isolated from sandflies (Diptera, Phlebotomidae) in Yugoslavia. Acta Biol Jug Mikrobiol. 1981;18:1–10.

34. Miščević Z, Gligic A, Vesenjak-hırjan J, Calisher CH, Miluninovic M, Biševac L. The association of sandflies (Diptera, Phlebotomidae) in the transmission of arboviruses in 2 areas of Yugoslavia. Acta Vet (Beogr). 1991;4:205–10.
